# Evaluation of Arecoline Hydrobromide Toxicity after a 14-Day Repeated Oral Administration in Wistar Rats

**DOI:** 10.1371/journal.pone.0120165

**Published:** 2015-04-16

**Authors:** Xiaojuan Wei, Jiyu Zhang, Jianrong Niu, Xuzheng Zhou, Jianyong Li, Bing Li

**Affiliations:** 1 Key Laboratory of New Animal Drug Project of Gansu Province, Gansu Province, China; 2 Key Laboratory of Veterinary Pharmaceutical Development of Ministry of Agriculture, Gansu Province, China; 3 Lanzhou Institute of Husbandryand Pharmaceutical Sciences of CAAS, Jiangouyan, Qilihe District, Lanzhou 730050, PR China; Weizmann Institute of Science, ISRAEL

## Abstract

A subchronic toxicity test was conducted in rats on the basis of a previous acute toxicity test to evaluate the safety of arecoline hydrobromide (Ah), to systematically study its pharmacological effects and to provide experimental support for a safe clinical dose. Eighty rats were randomly divided into four groups: a high-dose group (1000 mg/kg), medium-dose group (200 mg/kg), low-dose group (100mg/kg) and blank control group. The doses were administered daily via gastric lavage for 14 consecutive days. There were no significant differences in the low-dose Ah group compared to the control group (P>0.05) with regard to body weight, organ coefficients, hematological parameters and histopathological changes. The high-dose of Ah influenced some of these parameters, which requires further study. The results of this study indicated that a long-term, continuous high dose of Ah was toxic. However, it is safe to use Ah according to the clinically recommended dosing parameters. The level of Ah at which no adverse effects were observed was 100 mg/kg/day under the present study conditions.

## Introduction

Arecoline is an alkaloid extracted from areca nuts (*Areca catechu* L.)[1]. Arecoline and its derivatives have been used to treat Alzheimer's disease[2], diarrhea, intestinal worms and other gastrointestinal disorders, gonorrhea, visual ailments, fever, dysentery, hysteria, schizophrenia, headaches, dental disorders, octopus bites and elephant diseases and have also been used as abortifacients[3]. One study demonstrated that the alkaloids and polyphenols from arecanuts could be used to enhance the healing of burn wounds, leg ulcers and skin graft surgery[4]. In addition, arecoline has demonstrated various pharmacological activities, such as hypoglycemic activity[5], vascular relaxation[6] and molluscicidal activity[7,8].

Parasites, especially parasites found in the digestive system, can cause significant harm to livestock, resulting in low productivity, low fertility and, in some cases, death. To date, only a few types of deinsectization drugs are available in China and have been repeatedly used, which has led to drug resistance. Therefore, it is critical to develop a deinsectization drug with good efficiency and low drug resistance. Arecoline is highly effective against cysticercus in vitro[9]and has an inhibitive effect on *Fasciola hepatica*[10]. However, the arecoline content in *Areca catechu L*.extracts ranges from only 0.3% to 0.7%, which, coupled with the low efficiency of traditional extraction methods, greatly limits its clinical application. Additionally, while arecoline as a liquid is not convenient to administer, its salt is appropriate for clinical use. The proposed synthetic process can achieve a yield of more than 78%. The benefits of this new technology include a high yield, the easy purification of the product, a simple operation process, and easy scale-up for industrial production[11].

Because Ah is being developed for the treatment and prevention of parasitic diseases, it is important to characterize its subchronic toxicity in animals following 14 consecutive days of oral administration. The present study aimed to assess the subchronic toxicity of Ah, specifically after daily oral administration for 14 consecutive days in rats. Blood and tissue samples were collected at various time points for hematology, clinical chemistry and pathology analyses. As an evaluation of preclinical safety, this study will provide guidance for the design of further preclinical toxicity studies and clinical trials of Ah.

## Materials and Methods

### Chemicals and reagents

Arecoline hydrobromide(methyl 1,2,5,6-tetrahydro-1-methyl-3-pyridinecarboxylate hydrobromide, Ah) transparent crystals (purity: 99.5% by RE-HPLC)were prepared at the Lanzhou Institute of Husbandry and Pharmaceutical Sciences of CAAS(Lanzhou, China).

### Animals

The study was approved by the Ethics Committee of Animal Experiments at the Institute of Husbandry and Pharmaceutical Sciences of CAAS in Lanzhou, China. Eighty Wistar rats of both sexes, with a clean grade (Certificate No.: SCXK (Gan)2008-0075)and an initial body weight of 150–160 g, were purchased from the animal breeding facilities at Lanzhou University (Lanzhou, China). The animals were individually housed to allow recording of individual food consumption and to avoid bias from hierarchical stress. The animals were kept in plastic Macrolon cages (Suzhou Fengshi Laboratory Animal Equipment Co., Ltd, Huangqiao town city Suzhou, China) of an appropriate size with a stainless steel wire cover and chopped bedding. The light/dark cycle was 12/12 h, the temperature was 22±2°C and the relative humidity was 55±10%. Standard compressed rat feed from the animal breeding facilities at Lanzhou University and drinking water were supplied *ad libitum*. This study was conducted in strict accordance with the recommendations in the Guide for the Care and Use of Laboratory Animals of the National Institutes of Health. All surgeries were performed under sodium pentobarbital anesthesia, and all efforts were made to minimize suffering. The animals were allowed a 2-week quarantine and acclimation period prior to the start of the study.

### Dosing

The selected high, medium and low doses were 1000, 200 and 100 mg/kg bw, respectively, based on the results of acute toxicity testing and preliminary studies in rats. Ah was dissolved in distilled water in blackpaper-covered vials and aseptically administered to each rat intragastrically once per day based on the individual daily body weight for 14 consecutive days. The rats were randomly assigned to four groups: three test groups (n = 20 rats per group, male:female = 1:1) and a vehicle group that functioned as the control group (n = 20 rats, male:female = 1:1).

### Study design

Once exposure was initiated, the animals were inspected daily regarding their general condition and any clinical abnormalities. The bedding was changed daily, at which time the rats were submitted to in-hand observations, including their reactions to being handled, and their body weight and food consumption were recorded.

At the end of the drug administration period, the rats in each group were euthanized via exsanguination of the femoral artery and were then necropsied. During necropsy, blood was collected, and the following organs were dissected: liver, spleen, thymus, heart, lungs, stomach, duodenum, ileum, jejunum, colon, cecum, ovaries, uterus, kidney, adrenals, brain and testes. All organs were visually inspected and weighed directly either after dissection or after fixation to reduce mechanical damage. All organs were fixed in standard formalin for further histological processing based on previously described methods[12].

Hematology, clinical biochemistry, the visceral index and histopathology were analyzed at the end of the drug administration period. A portion of each blood sample was treated with EDTA-Na_2_ and analyzed for hematological indices, including red blood cellcount (RBC), white blood cellcount (WBC), lymphocyte count (LYM), granulocyte count (GRA), hemoglobin (HGB), hematocrit (HCT), mean corpuscular volume (MCV), mean corpuscular hemoglobin (MCH), mean corpuscular hemoglobin concentration (MCHC), platelet count (PLT), platelet distribution width (PDW), mean platelet volume (MPV), thrombocytocrit (PCT)and platelet larger cell ratio (P-LCR), using a hematology analyzer (Cell Dyn1200, Abbott, USA). In addition, an automatic biochemistry analyzer (XL-640, ERBA, Germany) was used to examine the sera obtained from the remaining blood samples for alanine aminotransferase (ALT), aspartate aminotransferase (AST), alkaline phosphatase (AKP), total protein (TP), albumin (ALB), globulin (GLO), total bile acid (TBA), blood urea nitrogen (BUN), creatinine (CREA), glucose (GLU), triglyceride (TG), cholesterol(CHOL) and lactate dehydrogenase (LDH).

### Histopathology

All tissues collected during necropsy were fixed in 10% neutral buffered formalin. Following fixation (and subsequent weighing, vide supra), the organs sampled for histological examination were dehydrated, paraffinized and embedded according to standard sampling and trimming procedures. Four-micron sections were stained with hematoxylin and eosin using an automated method. Microscopic observations were performed by an initial unblinded comparison of the control and high-dose samples. Blind and/or semi-quantitative scoring was used when changes were detected in the initial inspection.

### Statistics

Continuous data, including body weight, food consumption, hematology, blood biochemistry, and organ weights, are expressed as the mean±standard deviation. The differences in the ratios of the organ weight to the body weight were analyzed using either ANOVA with LSD or Dunnett’s test (SPSS 21.0 software, Chicago, IL, USA). Other data were analyzed using a repeated measures ANOVA built into ageneral linear model (SPSS 21.0). Inter-group comparisons were conducted using a multivariate general linear model. P-values <0.05 were considered statistically significant. For non-continuous data and comparisons of histopathological changes, statistical tests were ranked and examined using the Kruskal—Wallis test. If significant, the Wilcoxon rank sum test was applied for comparison with the control group. The male and female rats were separately evaluated.

### Observations of the clinical curative effect

To observe the clinical curative effect, 252 infected dogs were treated orally with the recommended doses of arecoline hydrobromide 15–24 hours after fasting. Within 72 h of administration, stool samples were selected and examined using the saturated salt solution float method to check polypides.

## Results

### In vivo observations

No deaths were observed in any group during the administration period. Compared to the control group, rats treated with Ah showed a decrease in some responses, including water and food consumption, and the male rats consumed more food than the female rats in all groups.

All of the rats in the test groups exhibited a significant reduction in body weight gain compared to the control animals (P<0.01; Table 1). The test group rats had significantly lower body weights during Ah administration, which indicated that long-term and continuous dosing had a toxic effect on the rats (Table 1). Interestingly, the effects in the female rats were more obvious than those in the male rats. There were no further clinical anomalies, and the dosing was well tolerated.

**Table 1:** Body weight changes (mean ± standard deviation) in male and female rats before and after Ah administration.

Group	Sex	Changes in body weight (g)
**Control**	Male	56.60±5.03
	Female	45.20±13.72
**100 mg/mL**	Male	43.70±4.00**
	Female	22.20±4.71**
**200 mg/mL**	Male	42.78±5.54**
	Female	20.90±10.66**
**1000 mg/mL**	Male	41.80±5.88**
	Female	19.98±13.72**

Note: Significant difference compared with male controls,

**P<0.01

### Hematology

According to the hematological analysis(Tables 2 and 3), the hematocrit and HGB levels and the leukocyte, lymphocyte and erythrocyte countsin female rats at all doses were significantly decreased following 14 days of Ah administration compared to the control rats (P<0.01). However, in the male rats, no significant changes in these indices were observed, with the exception of the HGB level in the high-dose group(P>0.05).

**Table 2:** Hematological parameters (mean ± standard deviation) in male rats intragastrically administered Ah daily for 14 consecutive days.

Dose	Control	100 g/kg	200 mg/kg	1000 g/kg
**No. of animals examined**	10	10	10	10
**RBC (10^12^/L)**	5.92±0.51	6.19±0.84	6.28±0.53	6.36±0.59
**WBC(10^9^/L)**	3.25±0.83	3.29±1.20	3.56±0.86	2.93±0.84
**GRA(10^9^/L)**	0.01±0.01	0.07±0.05*	0.07±0.05*	0.03±0.03
**LYM(10^9^/L)**	2.99±0.84	4.48±1.21*	3.74±0.58	2.38±0.70
**MID(10^9^/L)**	0.25±0.03	0.74±0.20*	0.75±0.27*	0.52±0.31
**MCV(fL)**	73.25±1.65	70.13±0.85**	72.34±1.01	71.11±1.34*
**HCT(%)**	43.43±4.78	43.46±6.38	45.44±3.91	38.17±4.52
**HGB(g/L)**	187.00±4.58	181.30±16.57	189.89±9.55	156.00±17.08**
**MCH(pg)**	433.16±35.32	420.21±24.56	419.27±20.19	409.47±11.60
**MCHC(g/L)**	31.69±1.90	29.45±1.44**	30.32±1.32	29.11±0.71**
**PLT(10^9^/L)**	1190.00±76.18	972±180.60*	1159.89±123.12	1017.90±66.70
**MPV(fL)**	7.63±0.14	7.93±0.50	7.83±0.29	7.66±0.31
**PDW(%)**	16.17±0.49	15.32±1.40	15.56±0.89	16.15±1.03
**PCT(%)**	0.91±0.53	0.77±0.15	0.90±0.09	0.78±0.07
**P-LCR(%)**	17.73±1.45	19.70±3.42	19.31±2.02	17.91±2.23

Note: Compared with control,

**P<0.01;

*P<0.05.

**Table 3:** Hematological parameters (mean ± standard deviation) in female rats intragastrically administered Ah daily for 14 consecutive days.

Dose	Control	100 g/kg	200 mg/kg	1000 g/kg
**No. of animals examined**	10	10	10	10
**RBC (10^12^/L)**	6.53±0.51	5.64±0.34*	5.22±0.83**	5.20±0.42**
**WBC(10^9^/L)**	8.35±1.22	4.19±1.16**	2.97±1.36**	2.92±0.70**
**GRA(10^9^/L)**	0.07±0.04	0.05±0.05	0.04±0.06	0.03±0.04
**LYM(10^9^/L)**	7.77±1.04	2.43±1.00**	3.64±1.19**	2.48±0.61**
**MID(10^9^/L)**	0.51±0.14	0.49±0.18	0.51±0.26	0.41±0.14
**MCV(fL)**	71.18±0.52	70.22±1.10	70.25±0.83	70.95±0.63
**HCT(%)**	46.50±3.56	36.66±5.86**	39.62±2.38*	36.90±3.15**
**HGB(g/L)**	200.67±9.29	172.40±7.75**	159.30±21.06**	148.90±10.58**
**MCH(pg)**	432.24±14.80	436.27±18.14	435.50±9.62	401.07±0.25*
**MCHC(g/L)**	30.77±1.16	30.63±1.30	30.59±0.76	28.66±0.70**
**PLT(10^9^/L)**	974.00±93.55	949.20±238.16	1124.20±67.75	786.70±175.63
**MPV(fL)**	7.80±0.32	7.75±0.48	7.91±0.15	7.95±0.21
**PDW(%)**	15.63±1.04	15.88±1.47	15.29±0.48	15.16±0.62
**PCT(%)**	0.76±0.51	0.73±0.19	0.89±0.53	0.63±0.13
**P-LCR(%)**	20.06±2.35	18.81±3.54	19.64±1.06	20.23±1.56

Note: Compared with control,

**P<0.01;

*P<0.05

### Blood biochemistry

The blood chemistry results are shown in Tables 4 and 5. Both female and male rats exhibited markedly decreased CHOL and AST in the high-dose group compared to the control animals(P<0.05). AKP was increased in the medium- and high-dose groups compared to the control animals, and the levels of AKP in the medium- and high-dose groups were significantly increased in both sexes after 14 consecutive days of dosing compared to the pre-dosing levels (P<0.05 and P<0.01, respectively).

**Table 4:** Biochemical parameters (mean ± standard deviation) in male rats intragastrically administered Ah daily for 14 consecutive days.

Dose	Control	100 mg/kg	200 mg/kg	1000 mg/kg
**No. of animals examined**	10	10	10	10
**ALT(U/L)**	68.56±1.53	67.89±6.72	70.78±5.21	70.78±12.20
**AST(U/L)**	303.70±31.75	307.50±22.07	298.70±33.64	261.60±53.98*
**AKP (U/L)**	118.50±10.10	118.40±10.94	138.70±9.83**	141.80±12.67**
**TP(g/L)**	71.03±0.76	70.58±2.59	71.41±3.22	80.53±7.28**
**ALB(g/L)**	37.80±1.39	36.38±2.66	36.09±1.78	35.67±2.32*
**GLO(g/L)**	32.39±1.39	33.15±1.65	33.24±2.70	39.34±6.34*
**TBA (μmol/L)**	26.47±0.32	27.84±5.06	32.75±7.17	48.74±15.90**
**BUN(mmol/L)**	19.67±14.77	15.03±5.21	13.23±3.16	10.41±2.15*
**CREA (μmol/L)**	81.33±102.17	35.44±34.06	30.59±27.31	14.37±2.84
**GLU (mmol/L)**	0.77±0.12	1.17±0.35	1.30±0.55**	1.48±0.44**
**TG (mmol/L)**	0.92±0.66	0.96±0.11	0.97±0.25	1.14±0.18**
**CHOL(mmol/L)**	2.42±0.18	2.33±0.22	2.09±0.28**	1.64±0.18**
**LDH (U/L)**	1285.67±167.50	1364.30±303.92	1193.00±151.99*	1151.10±123.84*

Note: Compared with control,

**P<0.01;

*P<0.05.

**Table 5:** Biochemical parameters (mean ± standard deviation) in female rats intragastrically administered Ah daily for 14 consecutive days.

Dose	Control	100 mg/kg	200 mg/kg	1000 mg/kg
**No. of animals examined**	10	10	10	10
**ALT (U/L)**	74.30±4.62	76.60±11.76	73.80±6.09	71.10±17.99
**AST(U/L)**	361.70±50.10	345.30±49.72	340.50±50.10	315.20±28.53*
**AKP (U/L)**	104.40±10.94	109.60±17.65	119.40±12.67*	123.00±19.37*
**TP (g/L)**	77.09±0.70	75.51±4.43	71.16±3.74*	70.58±2.30*
**ALB(g/L)**	37.50±0.61	36.17±2.39	37.92±3.40	38.19±2.68
**GLO(g/L)**	40.72±1.11	39.34±3.62	33.24±2.52**	32.39±1.95**
**TBA (μmol/L)**	31.21±10.02	24.59±2.23	29.98±7.49	31.44±16.17
**BUN(mmol/L)**	14.30±2.89	13.99±1.27	14.17±1.34	12.59±3.28
**CREA (μmol/L)**	24.06±7.42	17.29±8.34	18.78±3.57	22.07±12.94
**GLU (mmol/L)**	2.30±0.50	1.11±0.35**	1.15±0.25**	1.17±0.30**
**TG (mmol/L)**	1.40±0.32	1.09±0.31	1.29±0.39	0.93±0.27*
**CHOL(mmol/L)**	2.09±0.14	2.09±0.25	2.10±.021	1.52±0.26**
**LDH (U/L)**	1238.00±163.89	1281.70±218.85	1175.90±203.26	1156.50±115.26

Note: Compared with control,

**P<0.01;

*P<0.05

TP, GLO, GLU, and TG levels were all altered in the high-dose groups in both sexes; however, in the males, these indicators were increased, whereas they were decreased in the females compared to the controls(P<0.05). ALB, BUN, and LDH levels were all decreased in the high-dose male group compared to the control group(P<0.05). However, in the females, no differences were observed between the three test groups and the control group (P>0.05). Only ALT and CREA showed no change in either sex in any test group compared to the control groups.

In addition, the levels of blood GLU in the male rats were significantly increased in the medium- and high-dose groups compared to the pre-dosing levels (P<0.01). In contrast, the levels of blood GLU in the females were significantly decreased in all of the test groups compared to the control group (P<0.01). The levels of CHOL in the high-dose group were significantly decreased in both sexes(P<0.01)compared to the control group. Thus, the changing trends of AKP and CHOL in both sexes were the same. The differences in the blood chemistry between males and females are difficult to explain.

### Organ weight

In the male rats, the weight of the liver and spleen increased following Ah administration in a dose-dependent manner, and a significant difference was observed between the high-dose group and the control group (P<0.05; Table 6). In the female rats, the weight of the liver, kidney and brain significantly increased following Ah administration (P<0.05; Table 7). The other organs did not exhibit any dose-dependent changes. Only the liver weight in the high-dose group was significantly increased in both sexes after 14 consecutive days of dosing compared to the control (P<0.01).

**Table 6:** Relative organ weight (mean±standard deviation) in males intragastrically administered Ah daily for 14 consecutive days.

Dose	Control	100 mg/kg	200 mg/kg	1000 mg/kg
**No. of animals examined**	10	10	10	10
**Liver (mg/g)**	0.034±0.000	0.035±0.0035	0.042±0.00	0.069±0.097**
**Kidney (mg/g)**	0.008±0.000	0.008±0.001	0.008±0.001	0.009±0.001**
**Adrenal gland (mg/g)**	0.0002±0.0000	0.0002±0.0000	0.0002±0.0000	0.0002±0.0000
**Spleen (mg/g)**	0.002±0.000	0.003±0.000*	0.003±0.000*	0.003±0.000*
**Testis (mg/g)**	0.012±0.002	0.012±0.002	0.012±0.003	0.012±0.001
**Brain (mg/g)**	0.013±0.001	0.013±0.001	0.013±0.001	0.013±0.002
**Heart (mg/g)**	0.005±0.000	0.005±0.001	0.005±0.001	0.005±0.001
**Thymus (mg/g)**	0.002±0.000	0.002±0.001	0.002±0.000	0.002±0.001
**Lung (mg/g)**	0.006±0.001	0.006±0.000	0.006±0.000	0.007±0.001*
**Testis (mg/g)**	0.012±0.002	0.012±0.002	0.012±0.003	0.012±0.001

Note: Compared with control,

**P<0.01;

*P<0.05

**Table 7:** Relative organ weights (mean ± standard deviation) in female rats intragastrically administered Ah daily for 14 consecutive days.

Dose	Control	100 mg/kg	200 mg/kg	1000 mg/kg
**No. of animals examined**	10	10	10	10
**Liver (mg/g)**	0.033±0.001	0.035±0.005*	0.036±0.018*	0.038±0.020**
**Kidney (mg/g)**	0.007±0.001	0.008±0.001*	0.008±0.001*	0.008±0.001*
**Adrenal gland (mg/g)**	0.0003±0.0000	0.0003±0.0000	0.0003±0.0000	0.0003±0.0000
**Spleen (mg/g)**	0.003±0.000	0.003±0.000	0.003±0.000	0.003±0.000
**Ovary (mg/g)**	0.000±0.000	0.001±0.001*	0.000±0.000	0.000±0.000
**Uterus (mg/g)**	0.002±0.000	0.002±0.000	0.002±0.001	0.002±0.002
**Brain (mg/g)**	0.012±0.001	0.014±0.001*	0.014±0.002*	0.014±0.001*
**Heart (mg/g)**	0.004±0.000	0.005±0.001	0.005±0.000	0.005±0.001
**Thymus (mg/g)**	0.003±0.000	0.003±0.001	0.003±0.000	0.002±0.001
**Lung (mg/g)**	0.006±0.000	0.006±0.001	0.007±0.001*	0.006±0.000

Note: Compared with control,

**P<0.01;

*P<0.05

### Histopathology

Microscopic examination of the organs was performed on animals from the four dose groups. The histopathological findings are summarized in Tables 8 and 9 and are shown in Figs 1–24. No abnormal changes in either the organs or tissues were observed in the low-dose groups compared to the control groups. These results demonstrated that Ah did not induce remarkable histopathological alterations in the low-dose animals.

**Table 8:** Histopathological findings for male rats treated with Ah for 14 days.

Organ	Findings	Dose			
		Control	Low	Medium	High
**Male**	No. of animals	10	10	10	10
**Liver**	Granular degeneration, slight	1	2	5	8
	Vacuolar degeneration, slight	0	1	4	8
	Hepatic congestion, slight	0	3	5	9
**Kidney**	Granular degeneration	1	1	5	10
	Necrosis,focal	0	1	6	10
	Congestion, moderate	0	0	2	10
**Heart**	Congestion, focal	0	0	2	10
	Myocardial degeneration	0	1	3	10
	edema	1	1	1	10
**spleen**	Edema,slight	1	1	3	10
	Necrosis, focal	0	1	4	8
**Brain**	Degeneration, nerve cells	0	0	2	8
	Edema	0	1	2	9
	Congestion	0	0	0	10
**Duodenum**	Inflammation with eosinophilic infiltration	0	0	3	10
**Cecum**	Inflammation with eosinophilic infiltration	0	0	5	10
**Colon**	Inflammation with eosinophilic infiltration	0	0	5	10

**Table 9:** Histopathological findings for female rats treated with Ah for 14 days.

Organ	Findings	Dose			
		Control	Low	Medium	High
**Female**	No. of animals	10	10	10	10
**Liver**	Granular degeneration, slight	1	3	6	8
	Vacuolar degeneration, slight	1	1	4	8
	Hepatic congestion, slight	0	2	5	9
**Kidney**	Granular degeneration	1	2	3	10
	Necrosis,focal	0	1	5	10
	Congestion, moderate	0	0	2	10
**Heart**	Congestion, focal	0	0	3	10
	Myocardial degeneration	1	1	4	10
	edema	0	1	1	10
**spleen**	Edema,slight	1	0	4	10
	Necrosis, focal	0	1	5	8
**Brain**	Degeneration, nerve cell	0	0	3	8
	Edema	0	1	2	9
	Congestion	1	2	4	10
**Duodenum**	Inflammation with eosinophilic infiltration	0	0	2	10
**Cecum**	Inflammation with eosinophilic infiltration	0	1	4	10
**Colon**	Inflammation with eosinophilic infiltration	0	0	3	10

**Fig 1:**
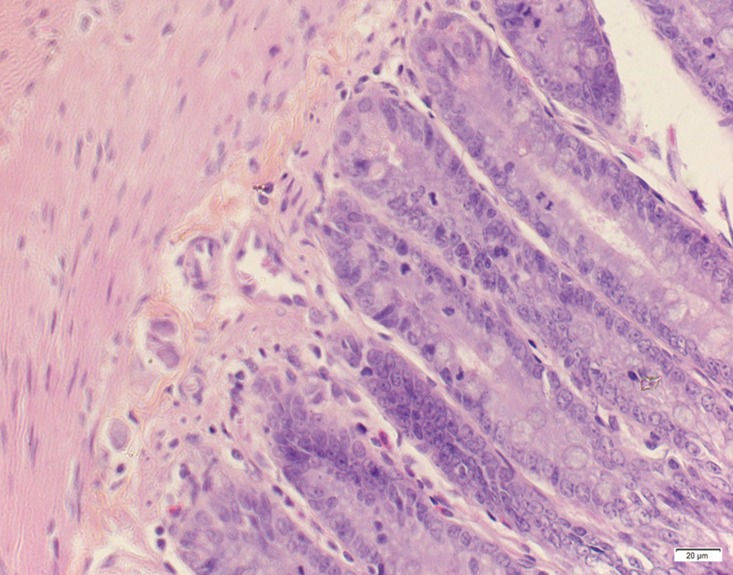
Photomicrograph of duodenum tissue of control group stained with HE. There is no abnormal.

**Fig 2:**
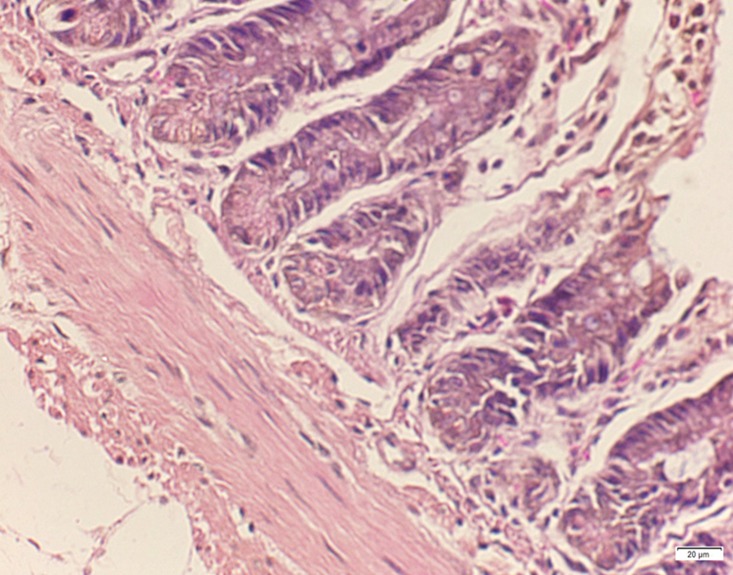
Photomicrograph of duodenum tissue of low dose group stained with HE. There is no abnormal.

**Fig 3:**
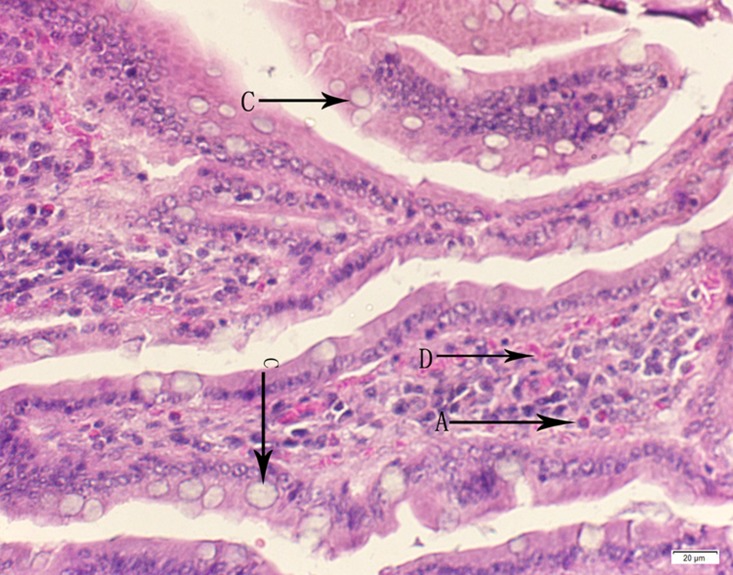
Photomicrograph of duodenum tissue of medium dose group stained with HE. A:Acute catarrhal enteritis with eosinophilic infiltration; C: Goblet cell hyperplasia; D: Congestion in the lamina propria.

**Fig 4:**
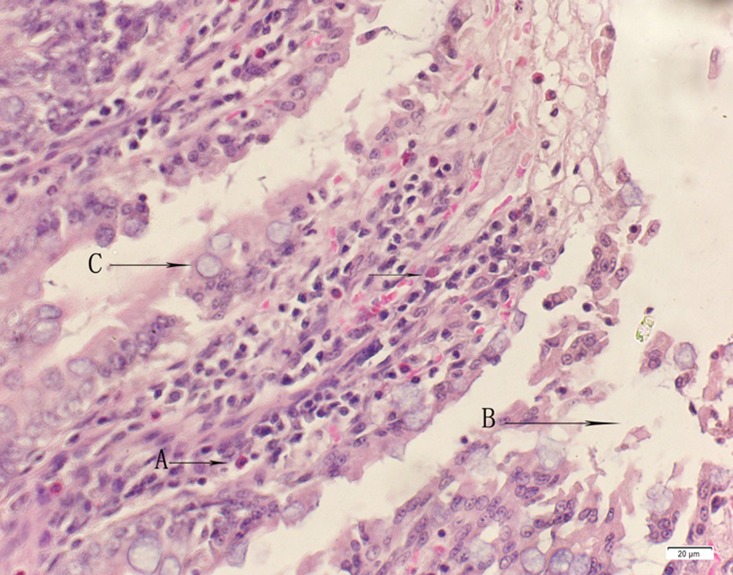
Photomicrograph of duodenum tissue of high dose group stained with HE. A:Acute catarrhal enteritis with eosinophilic infiltration; B:Degeneration, necrosis and exfoliation of intestinal epithelial cells; C: Goblet cell hyperplasia.

**Fig 5:**
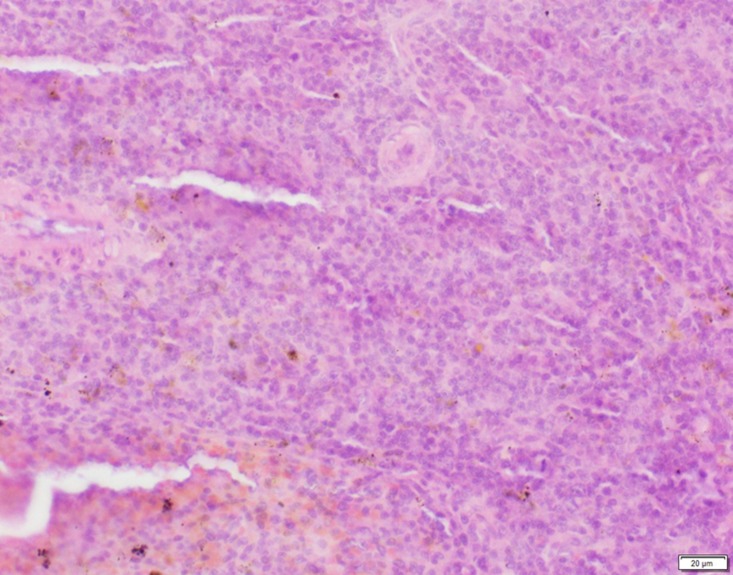
Photomicrograph of spleen tissue of control group stained with HE. There is no abnormal.

**Fig 6:**
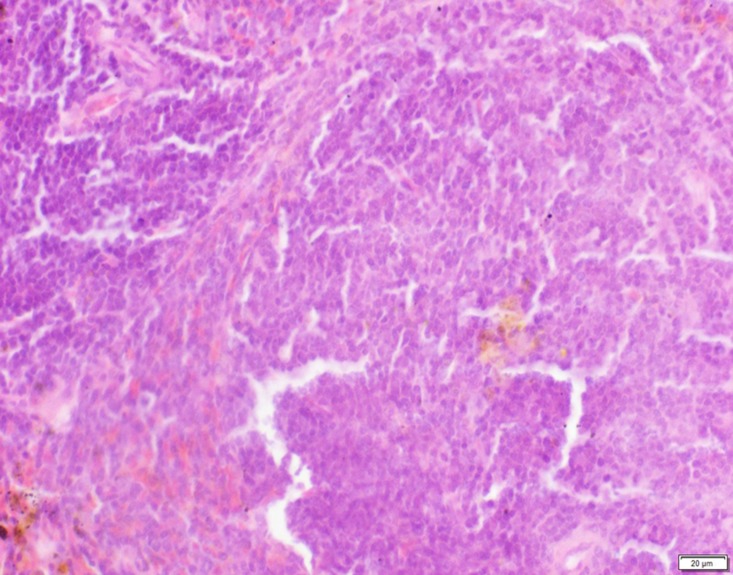
Photomicrograph of spleen tissue of low dose group stained with HE. There is no abnormal.

**Fig 7:**
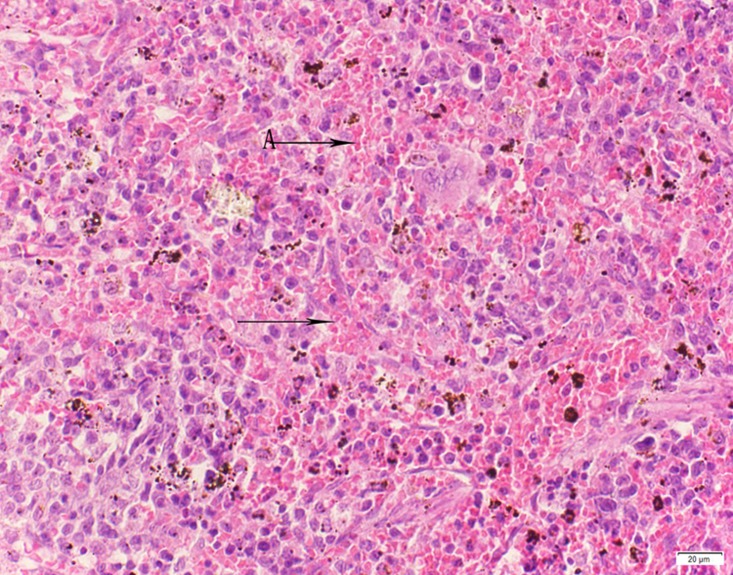
Photomicrograph of spleen tissue of medium dose group stained with HE. A: Passive congestion.

**Fig 8:**
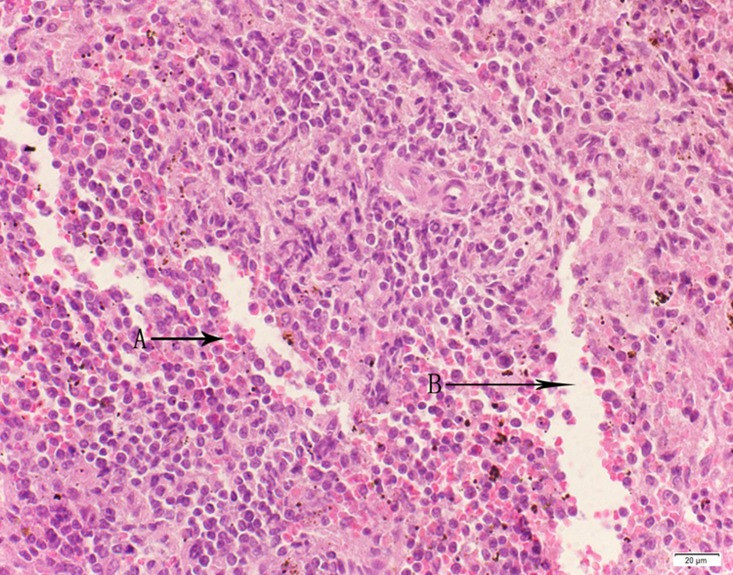
Photomicrograph of spleen tissue of high dose group stained with HE. A: Passive congestion; B: Edema.

**Fig 9:**
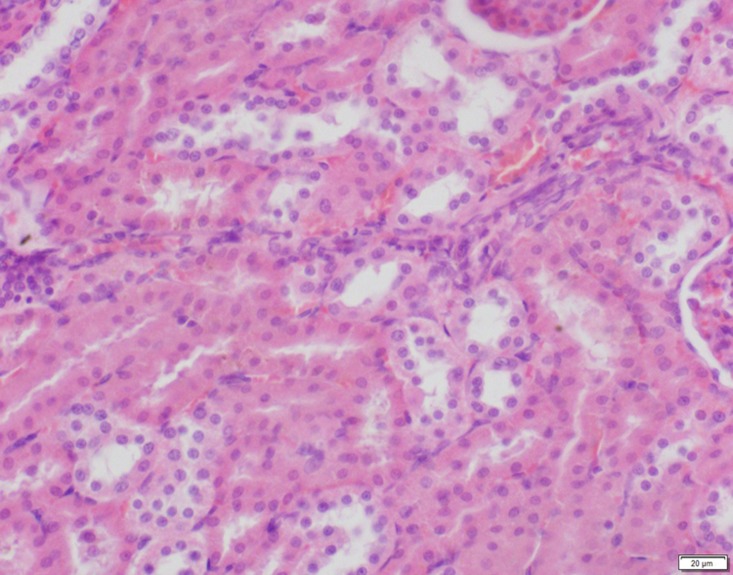
Photomicrograph of kidney tissue of control group stained with HE. There is no abnormal.

**Fig 10:**
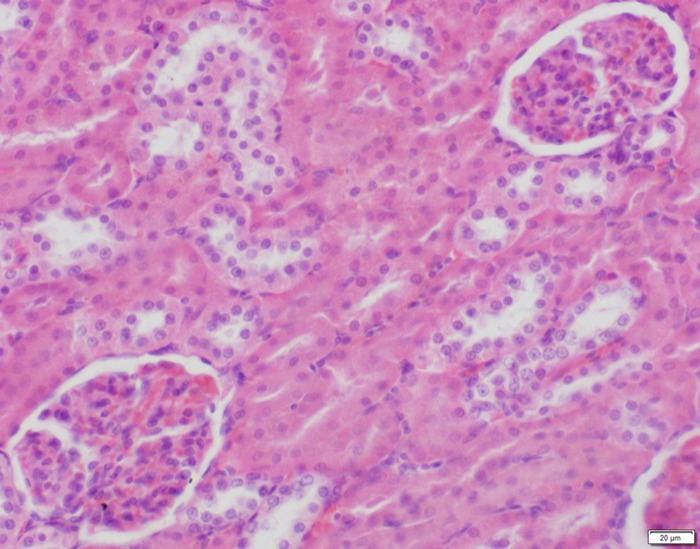
Photomicrograph of kidney tissue of low dose group stained with HE. There is no abnormal.

**Fig 11:**
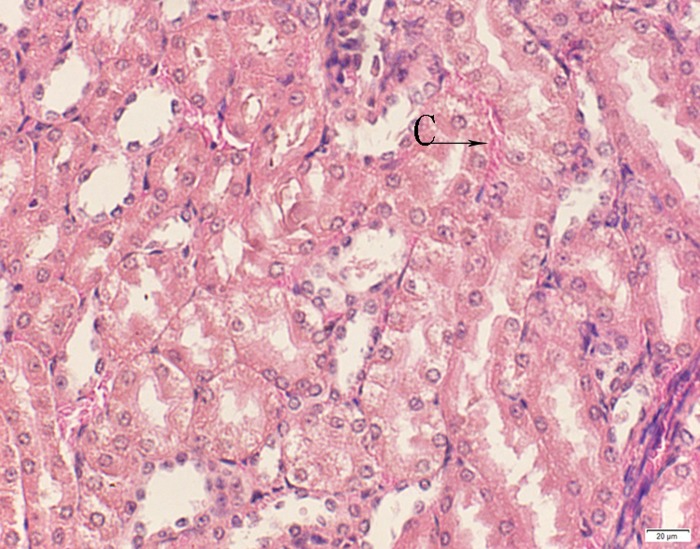
Photomicrograph of kidney tissue of medium dose group stained with HE. C: Congestion.

**Fig 12:**
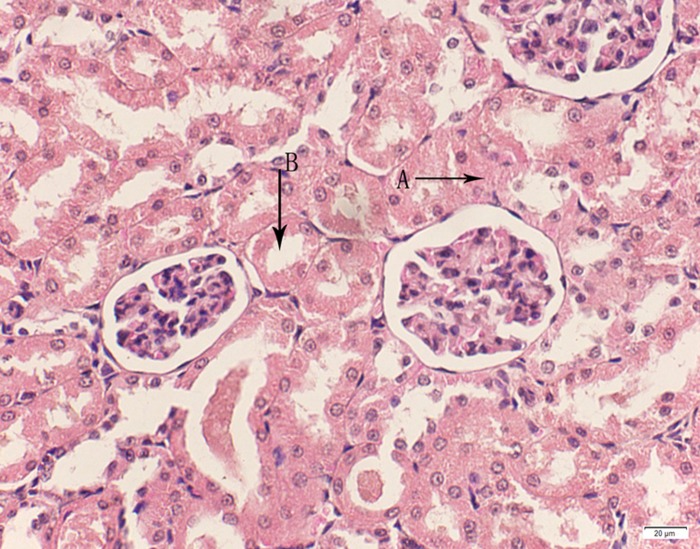
Photomicrograph of kidney tissue of high dose group stained with HE. A: Granular degeneration in renal tubular epithelial cells; B:Focalnecrosis.

**Fig 13:**
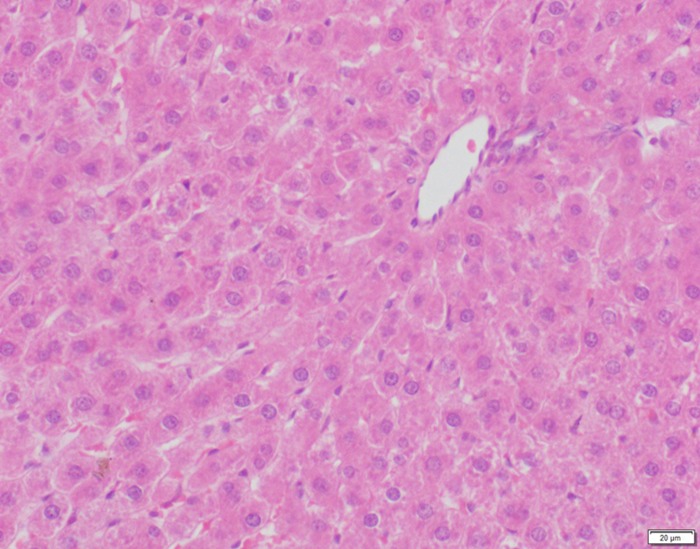
Photomicrograph of liver tissue of control group stained with HE. There is no abnormal.

**Fig 14:**
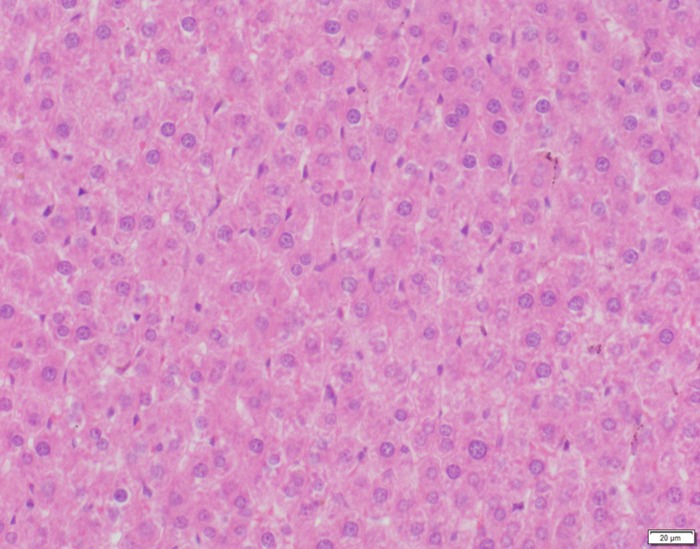
Photomicrograph of liver tissue of low dose group stained with HE. There is no abnormal.

**Fig 15:**
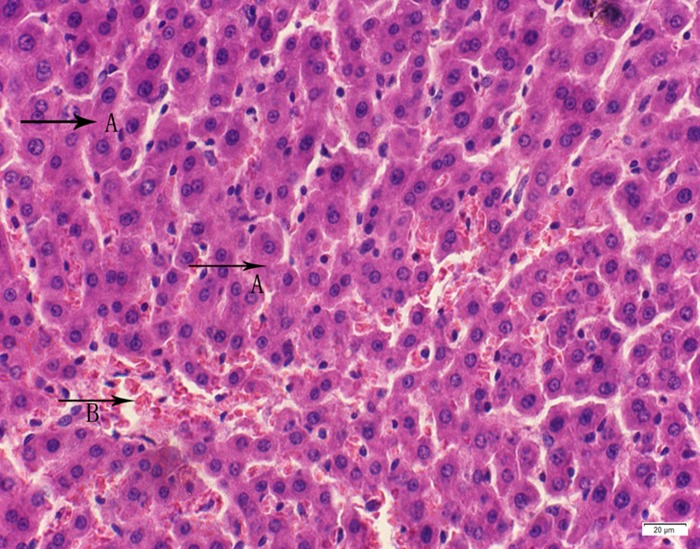
Photomicrograph of liver tissue of medium dose group stained with HE. A:Granular degeneration of liver cells; B:Congestion.

**Fig 16:**
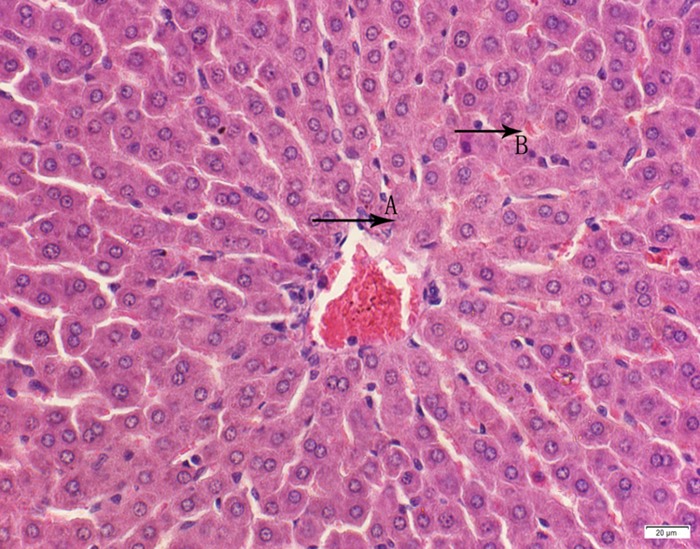
Photomicrograph of liver tissue of high dose group stained with HE. A:Granular degeneration of liver cells; B:Congestion.

**Fig 17:**
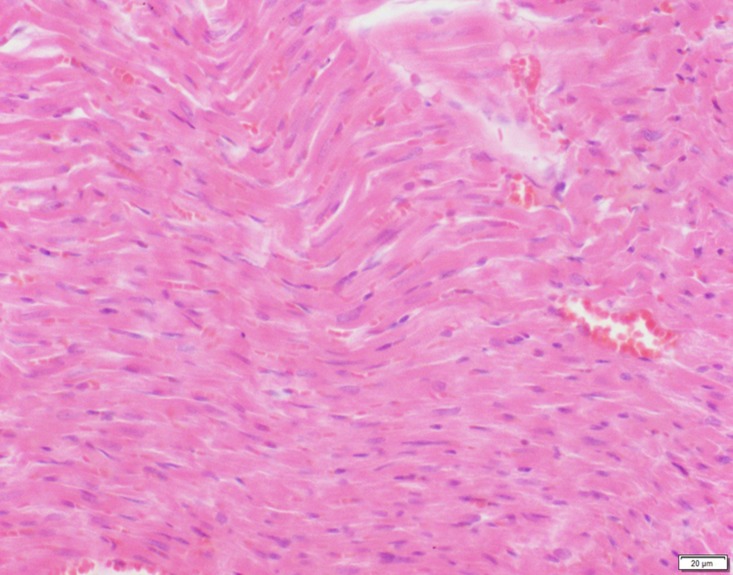
Photomicrograph of heart tissue of control group stained with HE. There is no abnormal.

**Fig 18:**
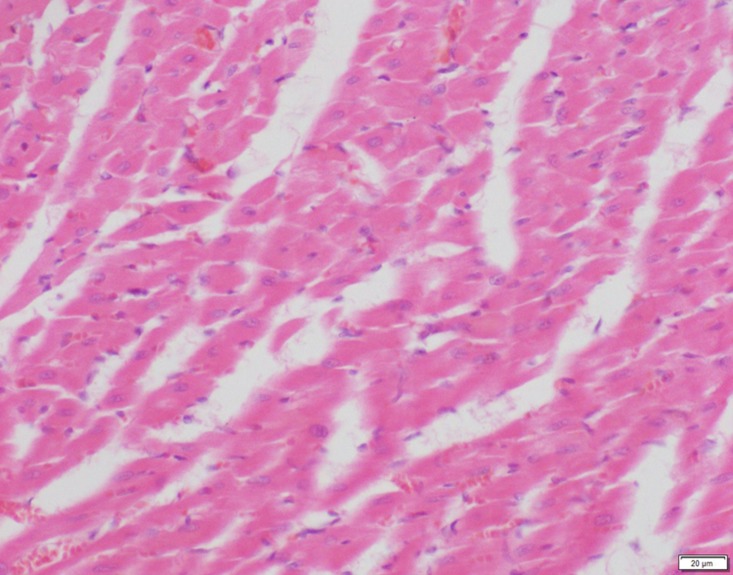
Photomicrograph of heart tissue of low dose group stained with HE. There is no abnormal.

**Fig 19:**
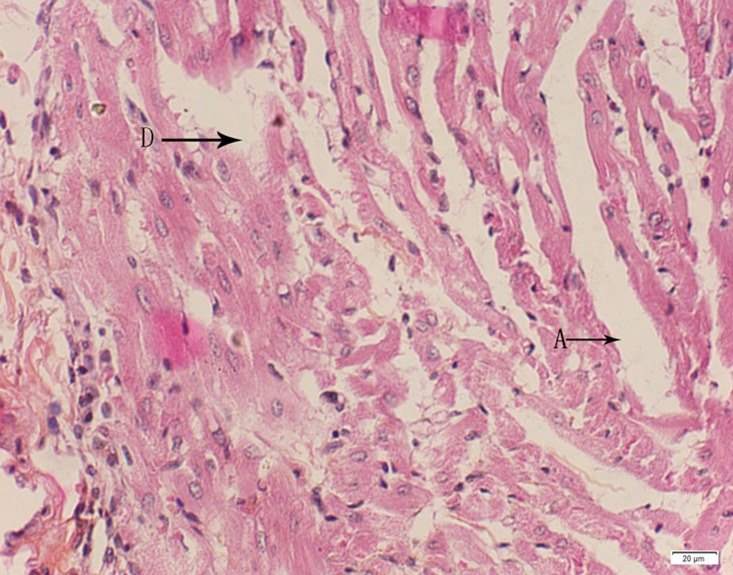
Photomicrograph of heart tissue of medium dose group stained with HE. A:Interstitial hydrops; D: Myocardial necrosis.

**Fig 20:**
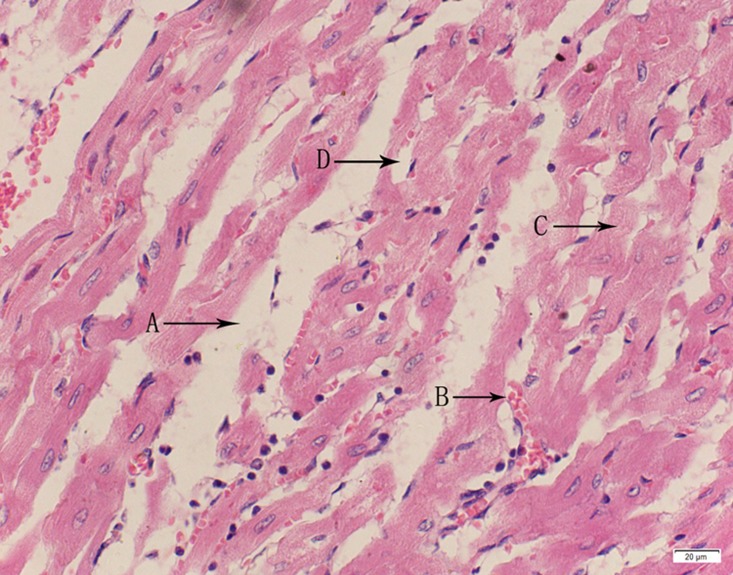
Photomicrograph of heart tissue of high dose group stained with HE. A:Interstitial hydrops; B: Congestion; C:Myocardial degeneration D: Myocardial necrosis.

**Fig 21:**
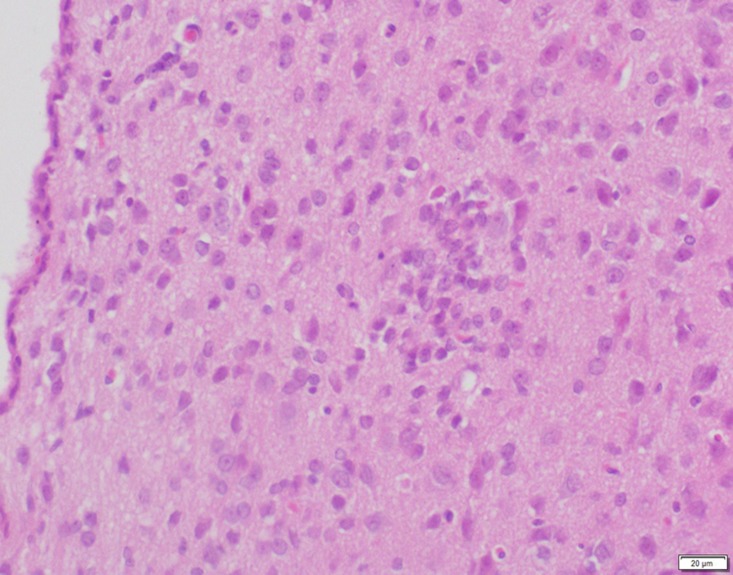
Photomicrograph of brain tissue of control group stained with HE. There is no abnormal.

**Fig 22:**
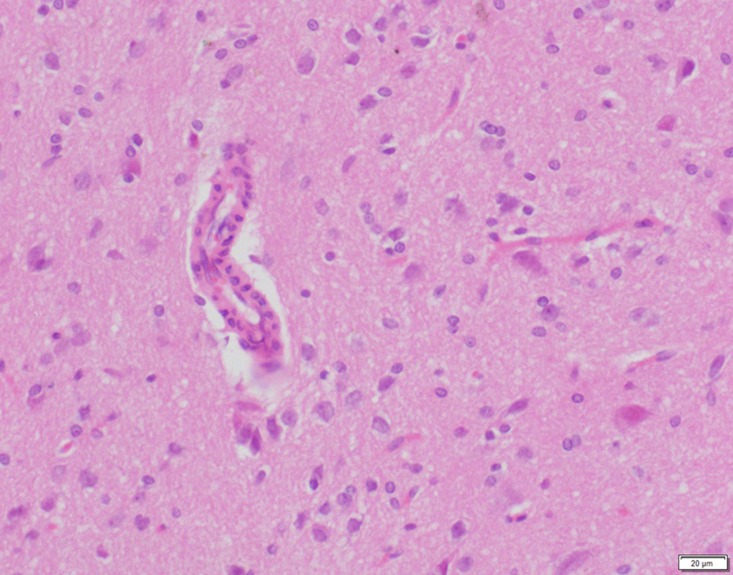
Photomicrograph of brain tissue of low dose group stained with HE. There is no abnormal.

**Fig 23:**
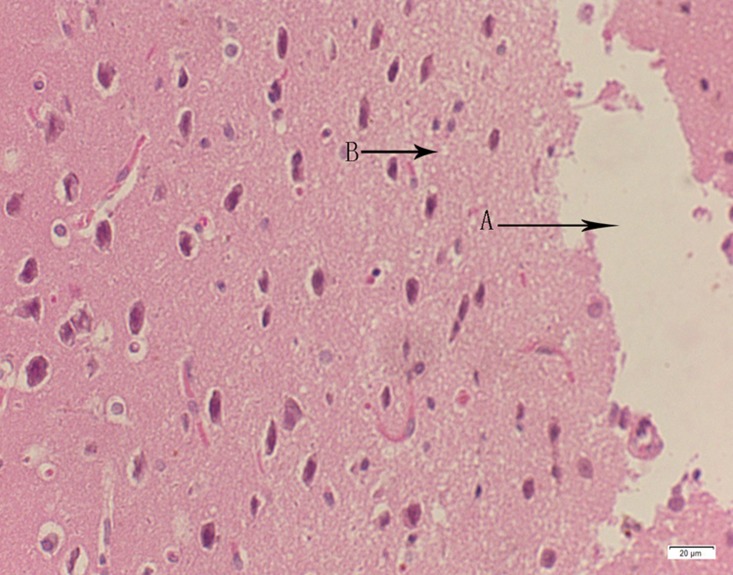
Photomicrograph of brain tissue of medium dose group stained with HE. A: Edema; B: Coagulative necrosis.

**Fig 24:**
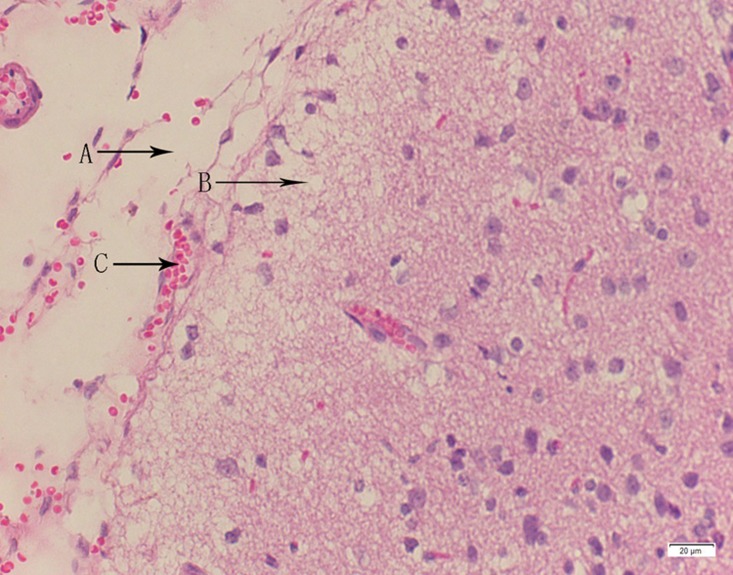
Photomicrograph of brain tissue of high dose group stained with HE. A: Edema; B: Coagulative necrosis; C: Congestion.

In the medium- and high-dose groups, treatment-related pathological findings were noted in the colon, spleen, heart, liver, kidney and brain. Acute catarrhal enteritis with eosinophilic infiltration was evident in 17 of the 20 rats in the high-dose group (Figs 1–4). Degeneration, necrosis and exfoliation of epithelial cells in the intestinal epithelial cells was observed, along with an increase in the number of goblet cells. In the spleens, the histopathological changes were characterized by slight degeneration, necrosis, and dropsy (Figs 5–8). The kidney presented moderate to severe granular degeneration in therenal tubular epithelial cells, focal necrosis and congestion in both genders (Figs 9–12). In the liver, hepatocellular damage was evident, including minor granule and vacuolar denaturation, necrosis and interstitial connective tissue proliferation (Figs 13–16). Myofibrillar tissue exhibited degeneration, necrosis and fibrinoid necrosis (Figs 17–20). Cellular necrocytosis was serious, the nuclei showed signs of karyolysis and were disappearing, and the cytoplasms were bursting. In the brain, mild congestion, edema and nerve cell degeneration were identified (Figs 21–24). There were statistically significant differences between the high-dose groups and control groups in terms of lesions.

### Observations of the clinical curative effect

Only worms with a scolex were regarded as a complete polypide. Among 252 infected dogs, 246 dogs were collected complete polypide. The species was identified according to Beveridge *et al* [13]. A total of six tapeworm species were identified: *Dipylidium caninum*, *Taenia hydatigena*, *Taenia pisiformis*, *Taenia multiceps*, *Echinococcus granulosus* and *Mesocestoides lineatus*.

## Discussion and Conclusion

Ah requires a deeper evaluation of its efficacy and safety because of its growing demand for reported medicinal use. We conducted a 14-day toxicity study to evaluate the safety of Ah at different doses. The selections of Ah dosage levels were primarily based on our preliminary test. Changes in body weight have been used as an indicator of adverse effects of drugs and chemicals[14]. Decreased body weight and food consumption were identified in all treated rats after 14 days of Ah administration. These findings suggest that oral Ah administration had some effect on the growth and functions of rats at the concentrations studied.

The hematopoietic system is one of the most sensitive systems used to assess drug toxicity in humans and animals[15]. The present study indicated that there were significant differences in hemoglobin, RBC, platelet, and total and differential leukocyte countsin the high-dose group, which indicates that Ah had specific effects on either the circulating blood cells or their production.

Liver damage results in the elevation of both ALT and AST levels in the blood. In addition, the identification of ALT in the serum is considered to be the first sign of cell and liver damage[16]. Creatinine is a good indicator of renal function, i.e., an increase in creatinine indicates there is obvious damage to functional nephrons. There were significant differences in ALP, ALT, AST, creatinine, blood urea, total bilirubin and total proteinlevels, as well as the A/G ratio, in the high-dose animals compared to the controls.

The liver is the site of cholesterol degradation and glucose synthesis, and it generates free glucose, which is secreted into the blood, from hepatic glycogen stores[17]. The subchronic toxicity test is one of the most important aspects of drug safety evaluation and the primary basis for the approval of a drug for clinical application. In general, continuous and repeated dosing is conducted to observe toxicity reactions as well as hematological, blood biochemical and pathological changes in experimental animals. It is also important to analyze the dose-toxicity relationship, the nature and degree of the toxicity reaction in the primary target organs, the reversibility of the toxicity reactions and the accumulation of toxicity. Compared to the control group, the rats in the high-dose group in this study exhibited weight loss and significantly increased liver and kidney organ coefficients.

The rats in the high-dose group were depressed and presented with a loss of appetite. Body weight change in experimental animals is a basic parameter that reflects apoisoning effect and the toxicity of the poison. The organ coefficient is an important index of the target organ affected by the test compound. In this study, the results indicated that the toxicity was lowest in the low-dose group; however, the toxicity increased with increasing doses of Ah.

Pharmacological studies can ascertain the toxicity of the test drug more precisely by determining whether the related physiological and biochemical parameters are altered under the effects of the tested drug. In this study, the rats in the high-dose group exhibited clear inhibitory effects on blood biochemical parameters, including ALT, TP and BUN levels. It was demonstrated that the toxicity reaction was intensified at the higher dosage, and clinical dosing should occur within a safe dosage range. A histopathological examination directly reflects drug toxicity. Our test results demonstrated that with a continuous increase in the Ah dose, organ damage was aggravated, with different degrees of congestion, degeneration and necrosis observed. The results accurately reflected the liver and kidney changes, whereas the results for the changes in the lung were poor. During the test, severe hemorrhage and congestion were observed in the livers of some rats, which could have been caused by damage due to an inappropriate lavage.

Based on the results of our analysis and the earlier acute toxicity test, long-term administration of high doses of Ah is likely to be toxic. The dosage should be set according to the clinically recommended dose to ensure safe dosing. This study provides a theoretical foundation for clinically safe dosing of Ah.
